# The risk for chronic kidney disease in patients with heart diseases: a 7-year follow-up in a cohort study in Taiwan

**DOI:** 10.1186/1471-2369-13-77

**Published:** 2012-08-03

**Authors:** Jiung-Hsiun Liu, Shih-Yi Lin, Chung-Yi Hsu, Hsin-Hung Lin, Chih-Chia Liang, Fung-Chang Sung, Chiu-Ching Huang

**Affiliations:** 1 Division of Nephrology and Kidney Institute, Department of Internal Medicine, China University Hospital, 2 Yuh-Der Road, Taichung City, 404, Taiwan; 2 Department of Pubic Health, China Medical University, 91 Hsueh-Shih Road, Taichung City, 404, Taiwan; 3 School of Medicine, China Medical University, Taichung, Taiwan; 4 Management Office for Health Data, China Medical University Hospital, Taichung, Taiwan

## Abstract

**Background:**

The worldwide increasing trend of chronic kidney disease (CKD) is of great concern and the role of heart disease deserves longitudinal studies. This study investigated the risk of developing CKD among patients with heart diseases.

**Methods:**

From universal insurance claims data in Taiwan, we retrospectively identified a cohort of 26005 patients with newly diagnosed heart diseases and 52010 people without such disease from the 2000–2001 claims. We observed prospectively both cohorts until the end of 2007 to measure CKD incidence rates in both cohorts and hazard ratios (HR) of CKD.

**Results:**

The incidence of CKD in the cohort with heart disease was 4.1 times greater than that in the comparison cohort (39.5 vs. 9.65 per 10,000 person-years). However, the HR changed into 2.37 (95% confidence interval (CI) = 2.05 – 2.74) in the multivariate Cox proportional hazard model after controlling for sociodemographic characteristics and comorbidity. Compared with individuals aged < 40 years, the HRs for CKD ranged from 2.70 to 4.99 in older age groups. Significant estimated relative risks of CKD observed in our patients were also independently associated with hypertension (HR = 2.26, 95% CI = 1.94 - 2.63) and diabetes mellitus (HR = 2.44, 95% CI = 2.13 - 2.80), but not with hyperlipidemia (HR =1.13, 95% CI = 0.99-1.30).

**Conclusions:**

This population study provides evidence that patients with heart disease are at an elevated risk of developing CKD. Hypertension and diabetes mellitus are also comorbidity associated with increasing the CKD risk independently.

## Background

The cardiac and renal diseases are always coexisting and may significantly increase mortality, other complications, and the cost of health care [1,2]. Heart diseases and chronic kidney disease (CKD) are thus often diagnosed and cared simultaneously in clinical practices. But, the nature of this association has not been well identified despite physicians’ efforts to take the clinical history in detail. The term “cardiorenal syndrome” (CRS) represents the concept of primary disorder of either the heart or kidney often results in secondary injury to each other. CRS has been well defined and classified but has not yet concluded a consensus process [3,4]. Studies have reported the interaction between chronic cardiac dysfunction and CKD, focusing the attention on the direction of primary CKD in the effect on heart disorders [5-10].

Although reports have provided data on CKD prevalence in heart diseases [11,12], several issues remain to be clarified. First, these study populations have been based on ethnic Caucasians and African Americans, and there are scant data on the ethnic Asian populations. Second, the subject number has been limited, cross-sectional design; fragmented appreciation of epidemiology, or the follow-up time has been relatively short.

Third, a further challenge in describing the epidemiology of CRS is that patients may be also in transition between acute and chronic condition at various time points. For these reasons, we need more evidence; knowing if this association between primary chronic heart dysfunction and subsequent development of CKD is real.

This study, a retrospective cohort study on ethnic Chinese, provides a unique opportunity to investigate the incidence of new-onset CKD among patients with pre-existing heart disease. The study subjects were of homogenous ethnicity. Longitudinal follow-up data made causal inference possible. We investigated whether heart disease has effect on subsequent development of CKD in this cohort.

## Methods

### Data source and study population

The Taiwan National Health Insurance (NHI), a universal health program established in 1995, has covered more than 96% of all 23 million people and has contracted with 90% of the hospitals and practitioners since 1996. We obtained the claims data of the Longitudinal Health Insurance Database established by the National Health Research Institute, Department of Health, Taiwan. This data contained the registry of a randomly selected one million insured people as of 2005. The claims data covered ambulatory care claims registry (CD), inpatient claims (DD) and the updated registry for beneficiaries (ID) in 1996–2007. We used the scrambled identification number to link data sets to safeguard the confidentiality of the insured population without ethical violation. This study was thus exempted from the ethical review.

### Study subjects

The study cohort of heart diseases consisted of new patients with at least 2diagnostic records of the heart disease in 2000–2001 based on The International classification of Disease, 9th Revision, Clinical Modification (ICD-9-CM) codes. They were rheumatic heart disease (ICD-9-CM 391, 393–398, and A-code 251), hypertensive heart disease (ICD 402), ischemic heart disease (ICD 410–414, and A270, A279) and others (ICD 420–429 and A281). The comparison cohort consisted of randomly selected people insured in 2000–2001 without heart diseases and frequency matched with age. We also excluded subjects with CKD at baseline (ICD-9-CM code 585), identified before the date subjects were selected for inclusion in the study. CKD is defined based on the glomerular filtration rate and/or abnormal serum creatinine concentration by the Taiwan Association of Nephrology which had established a registry system for CKD. The accuracy of diagnosis is the primary requirement for the registry system. Eventually, there were 26005 heart disease cases and 52010 references in this study. Patients with the baseline comorbidity including hypertension (ICD-9-CM 401–405 and A-code A260 A269), diabetes mellitus (ICD-9-CM 250 and A-code A181), and hyperlipidemia (ICD-9-CM 272 and A-code A182) were also identified. The Bureau of National Health Insurance conducted periodic review of claims data to ensure the accuracy of claims.

### Statistical analysis

Data analysis compared distributions of age, sex, occupation, residential area, income, and comobidity between the study cohort (subjects with heart disease) and the comparison cohort (subjects without heart disease), which were examined by Chi-square test. We calculated the incidence density rates of CKD by these variables and the corresponding study cohort to comparison cohort rate ratios of CKD. For incidence density calculation, we calculated follow-up person-years for study subjects until CKD diagnosed, or until 31 December 2007 for those uncensored, or the censoring date for the censored for other reasons, such as death, emigration and termination of the insured program. The same patient might have multiple admissions with different CKD stages. Only the first CKD event was used to estimate the follow-up person-years. Variables that were categorized included age (< 40, 40–49, 50–59 and ≥ 60 years), residential area (north, central, south, and east and off islands) and income [less than New Taiwan Dollar (NTD) 15,000, 15,000-29,999, and more than 30,000/ per month], urbanization level (population density) of residential township or district (high, moderate, and low) and occupation (white collar, blue collar, and others).We calculated hazard ratios (HRs) and 95% confidence interval (CI) using Cox hazard proportional model to assess the hazard ratio of CKD for patients with the heart disease. Two multivariate models were used by controlling categorical covariates. One model included sociodemographic variables with significant association. The other model included also baseline comorbidity. A plot of the Kaplan-Meier analysis was used to show the probability of persons remaining without CKD, and the log-rank test was used to test the difference between the study cohort and the comparison cohort. All analyses were performed by SAS statistical software (version 9.1 for Windows; SAS Institute, Inc., Cary, NC, USA). The hazard ratios are presented with 95 percent CIs, and p-values are two sides.

## Results

### Subjects characteristics

Table 1 compares distributions of sociodemographic characteristics and baseline comorbidity status between the two cohorts. Proportional distributions showed that there were more females in the study cohort than in the comparison cohort. Age, occupation and residential urbanization levels of study subjects were similar between the two cohorts. The distribution of residential regions and income were somewhat different but significant. The study cohort was more prevalent in comorbidity than the comparison cohort including diabetes mellitus (17.7% vs. 5.9%), hypertension (51.4% vs. 13.0%), and hyperlipidemia (27.3% vs. 8.8%).

**Table 1:** Comparisons in demographic characteristics and baseline comobidities between cohort of patients with heart disease and cohort without heart disease diagnosed in 2000-2001

**Variables**	**Heart Disease**						
	**No**		**Yes**^**†**^		**Total**		
	**N = 52010**		**N = 26005**		**N = 78015**		
	**n**	**(%)**	**n**	**(%)**	**n**	**(%)**	**p-value**
Sex							<0.0001
Female	24733	(47.6)	13829	(53.2)	38562	(49.4)	
Male	27277	(52.4)	12176	(46.8)	39453	(50.6)	
Age, years							1.00
< 40	11352	(21.8)	5676	(21.8)	17028	(21.8)	
40-49	10438	(20.1)	5219	(20.1)	15657	(20.1)	
50-59	10584	(20.4)	5292	(20.4)	15876	(20.4)	
≥ 60	19636	(37.8)	9818	(37.8)	29454	(37.8)	
Occupation							0.26
White collar	20386	(39.2)	10050	(38.7)	30436	(39.0)	
Blue collar	20785	(40.0)	10434	(40.1)	31219	(40.0)	
Others	10839	(20.8)	5521	(21.2)	16360	(21.0)	
Urbanization^†^							0.63
Low	7889	(15.2)	3954	(15.2)	11843	(15.2)	
Moderate	10167	(19.6)	5154	(19.8)	15321	(19.6)	
High	33954	(65.3)	16896	(65.0)	50850	(65.2)	
Region^†^							<0.0001
North	23081	(44.4)	11334	(43.6)	34415	(44.1)	
Central	9988	(19.2)	5641	(21.7)	15629	(20.0)	
South	14231	(27.4)	6691	(25.7)	20922	(26.8)	
East and Island	4710	(9.1)	2338	(9.0)	7048	(9.0)	
Monthly income (NTD)							<0.0001
<15,000	23332	(44.9)	11748	(45.2)	35080	(45.0)	
15,000-29,999	20072	(38.6)	10362	(39.9)	30434	(39.0)	
≥30,000	8606	(16.6)	3895	(15.0)	12501	(16.0)	
DM^‡^							<0.0001
No	48951	(94.1)	21392	(82.3)	70343	(90.2)	
Yes	3059	(5.9)	4613	(17.7)	7672	(9.8)	
Hypertension^‡^							<0.0001
No	45261	(87.0)	12628	(48.6)	57889	(74.2)	
Yes	6749	(13.0)	13377	(51.4)	20126	(25.8)	
Hyperlipidemia^‡^							<0.0001
No	47433	(91.2)	18901	(72.7)	66334	(85.0)	
Yes	4577	(8.8)	7104	(27.3)	27.3	(15.0)	

Heart Disease: ICD-9 coden (A-code): rheumatic 391, 393–398 (A251), hypertensive 402, ischemic 410–414 (A270, A279), else 420–429 (A281) and times ≥ 2.

Urbanization: low = 1st and 2nd quartile of density of population, moderate = 3rd quartile of density of population, high = 4th quartile of density of population.

Chronic Kidney Disease (CKD): ICD-9 coden: 585 and times ≥ 2 between 2001 and 2007.

Diabetes Mellitus (DM): ICD-9 coden (A-code): 250 (A181) and times ≥ 3.

Hypertension: ICD-9 coden (A-code): 401–405 (A260, A269) and times ≥ 3.

Hyperlipidemia: ICD-9 coden (A-code): 272 (A182) and times ≥ 3.

^†^1 observation of Heart Disease patients is missing.

^‡^The dates of diagnosis of DM, Hypertension, and Hyperlipidemia were before December 31, 2001.

### Risk and crude relative risk of CKD

Table 2 summarizes the CKD incidence densities in the observed follow-up period for the two cohorts, and study cohort to comparison cohort rate ratios. There were 714 cases of CKD identified in a follow-up of 180,736 person-years in the cohort with heart disease, and 352 cases of CKD in 364,887 person-years in the comparison cohort. The incidence rate ratio of CKD in the study cohort was 4.1 times higher than that in the comparison cohort (39.5 vs. 9.65 per 10,000 person-years). The rate ratios measured by the categorized sociodemographic status ranged 3.30 to 4.75. The study cohort less than 40 years of age had a much greater rate ratio of 10.7. The incidence rate ratios were 2.71 for subjects with diabetes mellitus, 2.26 for those with hypertension and 2.81 for those with hyperlipidemia.

**Table 2:** Incidence densities of chronic kidney disease in heart disease cohort and comparison cohort and rate ratios by sociodemographic characteristics and baseline comobidities

	**Heart Disease**							
	**No**				**Yes**			
	**Case**	**Person-year**	**Rate**^†^		**Case**	**Person-year**	**Rate**^**†**^	**Ratio**
All	352	364,887	9.65		714	180,736	39.51	4.10
Sex								
Female	125	173,553	7.20		311	96,370	32.27	4.48
Male	227	191,335	11.86		403	84,367	47.77	4.03
Age, years								
< 40	6	79,454	0.76		32	39,618	8.08	10.70
40-49	44	73,240	6.01		90	36,416	24.71	4.11
50-59	69	74,360	9.28		132	36,853	35.82	3.86
≥ 60	233	137,834	16.90		460	67,850	67.80	4.01
Occupation								
White collar	101	143,047	7.06		200	69,951	28.59	4.05
Blue collar	169	145,810	11.59		320	72,524	44.12	3.81
Others	82	76,030	10.79		194	38,261	50.70	4.70
Urbanization^‡^								
Low	65	55,349	11.74		134	27,496	48.73	4.15
Moderate	67	71,393	9.38		154	35,819	42.99	4.58
High	220	238,146	9.24		426	117,413	36.28	3.93
Region^‡^								
North	125	161,939	7.72		289	78,784	36.68	4.75
Central	63	70,161	8.98		157	39,248	40.00	4.45
South	121	99,742	12.13		186	46,492	40.01	3.30
East and Island	43	33,045	13.01		82	16,205	50.60	3.89
Monthly income (NTD)								
<15,000	187	163,776	11.42		386	81,452	47.39	4.15
15,000-29,999	135	140,693	9.60		280	72,062	38.86	4.05
≥30,000	30	60,418	4.97		48	27,222	17.63	3.55
DM^§^								
No	275	343,556	8.00		404	149,045	27.11	3.39
Yes	77	21,331	36.10		310	31,691	97.82	2.71
Hypertension^§^								
No	221	317,675	6.96		133	87,930	15.13	2.17
Yes	131	47,212	27.75		581	92,806	62.60	2.26
Hyperlipidemia^§^								
No	287	332,801	8.62		433	131,402	32.95	3.82
Yes	65	32,086	20.26		281	49,334	56.96	2.81

^†^per 10,000 person-year.

^‡^1 observation of Heart Disease patients is missing.

^§^The dates of diagnosis of DM, Hypertension, and Hyperlipidemia were before December 31, 2001.

### Hazards of CKD

The association of developing CKD was significantly greater in men than women and the HR increased with age in the multivariate Cox model (Table 3, model 2 and model 3). The HR of developing CKD was augmented with age after adjusted for socioeconomic factors (Table 3, model 2). The statistical significance still existed even after additional adjustment for cardiometabolic risks such as diabetes, hypertension and hyperlipidemia (Table 3, model 3). In univariate analysis, the HR of developing CKD for patients with heart disease was 4.10 (CI = 3.61-4.66) (Table 3, model 1). In multivariate models, the HRs were 4.20 (CI = 3.70-4.78) after adjusted for baseline sociodemographic factors (Table 3, model 2) and decreased to 2.37 (95% CI = 2.05 - 2.74) after adjusted for baseline sociodemographic and cardometabolic factors (Table 3, model 3). The risk of developing CKD was slightly greater for patients with comorbidity of diabetes mellitus (HR = 2.44, 95% CI = 2.13 to 2.80) than that of hypertension (HR = 2.26, 95% CI =1.94 to 2.63) (Table 3, model 3). Patients with hyperlipidemia had a moderate HR of 1.13 (95% CI = 0.99 to 1.30) to have CKD with statistically insignificant (p > 0.05).

**Table 3:** Hazard ratios for risk factors of chronic kidney disease

**Variables**	**Model 1**	**Model 2**	**Model 3**
	**HR (95% CI)**	**HR (95% CI)**	**HR (95% CI)**
Heart disease			
No	1.00 (reference)	1.00 (reference)	1.00 (reference)
Yes	4.10 (3.61-4.66)***	4.20 (3.70-4.78)***	2.37 (2.05-2.74)***
Sex			
Female		1.00 (reference)	1.00 (reference)
Male		1.50 (1.32-1.69)***	1.56 (1.38-1.77)***
Age			
< 40		1.00 (reference)	1.00 (reference)
40-49		3.79 (2.64-5.44)***	2.70 (1.87-3.88)***
50-59		5.32 (3.76-7.54)***	3.17 (2.22-4.52)***
≥ 60		9.23 (6.62-12.87)***	4.99 (3.55-7.03)***
Occupation			
White collar		1.00 (reference)	1.00 (reference)
Blue collar		1.22 (1.01-1.46)*	1.19 (0.99-1.43)
Others		1.01 (0.84-1.20)	0.99 (0.83-1.18)
Monthly income (NTD)			
<15,000		1.45 (1.11-1.90)**	1.41 (1.08-1.85)*
15,000-29,999		1.24 (0.94-1.64)	1.28 (0.97-1.69)
≥30,000		1.00 (reference)	1.00 (reference)
DM			
No			1.00 (reference)
Yes			2.44 (2.13-2.80)***
Hypertension			
No			1.00 (reference)
Yes			2.26 (1.94-2.63)***
Hyperlipidemia			
No			1.00 (reference)
Yes			1.13 (0.99-1.30)

Model 1: Cox hazard proportional analysis without adjustment.

Model 2: Multivariate Cox hazard proportional analysis including age, sex, occupation, and income.

Model 3: Multivariate Cox hazard proportional analysis including age, sex, occupation, income, diabetes, hypertension, and hyperlipidemia.

^†^One missing in model 2 and model 3.

*p < 0.05, **p < 0.01,***p < 0.001.

**Figure 1:**
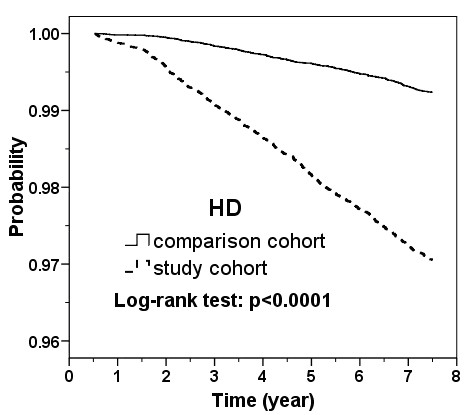
**Kaplan-Meier plot for probability of subjects free from chronic kidney disease in the follow-up period in patients with heart disease and comparison cohort without heart disease.** (HD = heart disease).

The plot of Kaplan-Meier analysis displayed that the CKD-free probability was 2.0 % lower in the study cohort than in the comparison cohort (97.3% vs. 99.3%, log-rank test: p < 0.0001) (Figure 1).

## Discussion

New onset of CKD is a frequent phenomenon in Taiwanese. The incidence rates of CKD are strongly related to diabetes mellitus and high blood pressure in the study subjects, particularly in the heart disease cohort. Among subjects without heart disease, those with diabetes mellitus were 4.5-fold (36.10 vs. 8.00 per 10,000 person-years) more likely to develop CKD than those without diabetes mellitus. The risk of developing CKD is particularly high for patients with both heart disease and diabetes mellitus, with the incidence increased to 97.82 per 10,000 person-years. The risk of developing CKD in patients with heart disease is followed by those with comorbidity of hypertension (62.60 per 10,000 person-years) and hyperlipidemia (56.96 per 10,000 person-years). Cardiovascular events play a critically important role in predicting CKD. The study results showed clearly a much higher risk of CKD among ethnic Chinese with underlying heart diseases and diabetes.

Unlike other studies, our study showed CKD incidence is not as high as physician’s suspicion in patients with heart diseases. This is due partly to the study design, which is related to complete exclusion of CKD patients at the study baseline in our cohort. This design decreases the possibility of the effect by a condition of mixture of cardiac and renal dysfunction at study baseline. This study has the advantage of a large sample of participants free of kidney disease at baseline, a long follow up period, and the ability to study the associated sociodemographic status at its inception. We are also able to study models composed of baseline comobidity. The models offer unique and complementary insights into risk factors for CKD. The baseline risk factor model is advantage because risk factors clearly precede outcome.

We have further measured the incidence and HR of developing CKD for different heart diseases, including rheumatic heart disease, hypertensive heart disease, ischemic heart disease, chronic heart failure and valvular heart disease. The incidence was the highest for those with chronic heart failure (68.1 per 10,000 person-years) with a HR of 3.08 (95% CI = 2.20-4.33), followed by hypertensive heart disease (62.6 per 10,000 person-years), ischemic heart disease (38.8 per 10,000 person-years), rheumatic heart disease (21.8 per 10,000 person-years) and valvular heart disease (11.8 per 10,000 person-years; HR, 1.36, 95% CI =0.89-2.10) (data not shown). Patients were most prevalent with ischemic heart disease accounted for 10600 persons.

The pathophysiological mechanisms underlying this reciprocal relationship between the heart and kidneys are still enigmatic. In general, the pathophysiologies of impaired renal function in cardiovascular disease are multifactorial and are associated with decreased renal perfusion, atherosclerosis and inflammation, endothelial dysfunction, and neurohormonal activation [13-15]. Our results suggest the importance of atherosclerotic risk factors for developing CKD and this is consistent. Moreover, additional analyses that we made provide us more information. Hypertensive heart disease, ischemic heart disease, and chronic heart failure, but not rheumatic or valvular heart disease, are the significant factors for developing CKD in patients with heart disease. These results remind us the importance of atherosclerotic risk factors, related heart disease, and chronic heart failure as their terminal stage, for developing CKD. Growing evidence suggests that atherosclerosis has direct effects on the kidney, largely because of intrarenal microvascular and glomerular disease that precedes the onset and represents the silent phase of ischemic renal disease [16-18]. Renal function abnormalities may exist at the early stages of atherogenesis and in patients with evidence of only extrarenal atherosclerosis and may precede the onset of overt ischemic nephropathy [18,19]. Indeed, nonobstructive atherosclerosis accelerates the decrease of renal size and the increased of serum creatinine level with age [18,20], implying that deterioration of renal function is likely the result of direct parenchyma compromise, likely provoked by atherogenic factors. Reduced cardiac output leads to hypoperfusion of the kidney as the result of poor forward flow is traditionally believed to be the main determinant of worsening renal function in patients with heart diseases [13].

The majority of patients with heart diseases may represent with increased central or peripheral congestion. Venous congestion might be one of the important factors for developing CKD. The presence of venous congestion has been considered as a secondary phenomenon due to the backward failure caused by impaired cardiac output. However, no evidence of association between left ventricle ejection fraction and estimated glomerular filtration rate can be consistently demonstrated in recent publication [21]. Patients with chronic heart failure and preserved left ventricle function appear to have similar estimated glomerular filtration rate than ones with impaired left ventricle function (ejection fraction less than 45%). Thus, it is uncertain whether the cardiac output still plays a crucial role in influencing the renal dysfunction. The impact of venous congestion, rather than cardiac output has risen in influencing CRS in recent years. It is recently considered is primarily associated with developing renal impairment in patients with advanced heart failure [22-24]. Transmission of venous congestion to the renal veins further impairs the glomerular filtration rate [24-28]. Mullens et al. reported that in patients with advanced decompensated heart failure, improvement of cardiac index after therapy had a limited contribution to worsening renal function [28]. The observations provide important clinical information that preservation of cardiac output without relieving venous congestion may not necessarily avert the development of renal impairment [27,28]. Increased central venous pressure and venous congestion also causes an increase in renal interstitial pressure, which might lead to a hypoxic status of renal parenchyma, similar to the mechanism by which hepatic congestion lead to hepatic dysfunction in heart failure [29-32].

This study had the limitations inherent in its retrospective and observational design. The severity of heart diseases and CKD was not quantified; thus, we were unable to measure whether the disease severity is associated with a dose–response relationship. In addition, the episodes of acute kidney injury during follow-up period might affect the accuracy of accumulated incidence in both study and control cohorts. Thus, our results regarding the association of heart diseases and CKD might be affected by referral bias. Moreover, physicians other than nephrologists might diagnose CKD by the definition of abnormal serum creatinine concentration. It is possible that some patients with earlier stages (stage 1–2) of CKD were not identified. Although this coding is somewhat less sensitive for identifying CKD in its early stage, it is used as a measure of kidney function by physicians other than nephrologists. Thus, the diagnostic values of patients with early stage CKD have not yet been fully evaluated. Further studies addressing this issue are warranted. Finally, we have not yet studied the long-term effect of time-averaged risk factors. Thus, it can not allow us to account the risk of CKD for changes in risk factors over time.

## Conclusions

Our data show that heart disease is tightly associated with increasing risk of subsequent development of CKD. It is mandatory to design a randomized controlled trial aimed at identifying pathophysiologically sound interventions targeting the relationship that we identify.

## Abbreviations

CKD, Chronic kidney disease; CRS, Cardiorenal syndrome; NHI, National health Insurance; ICD, International classification of disease; NTD, New Taiwan Dollar; HR, Hazard ratio; CI, Confidence interval.

## Competing interests

All authors have no conflicts of interests, or financial or other relationship to declare that may influence or bias this work.

## Authors’ contributions

Each author contributed to this manuscript. JHL analyzed the data and wrote the manuscript. HHL and CYH contributed substantially to the statistical analysis and interpretation of the data, and to the manuscript organization and editing. SYL and CCL contributed to the conception and design of the study and on-going progress of the study. FCS and CCH designed and revised this study. All authors reviewed and approved the manuscript.

## Pre-publication history

The pre-publication history for this paper can be accessed here:

http://www.biomedcentral.com/1471-2369/13/77/prepub
